# Rail Corrugation Detection and Characterization Using Computer Vision

**DOI:** 10.3390/s21248335

**Published:** 2021-12-13

**Authors:** Harris Lee, Jiyoung Hong, Tariku W. Wendimagegn, Heekong Lee

**Affiliations:** 1 Dermamirror Co., Ltd., Umyeon-dong 66-2, Seoul 809, Korea; harris@haanvision.com (H.L.); adetariku@gmail.com (T.W.W.); 2 Korea Railroad Research Institute, Cheoldobangmulgwan-ro 176, Uiwang-si 16105, Korea; hongjy@krri.re.kr

**Keywords:** rail corrugation detection, cepstrum transformation, frequency-domain analysis, static harmonic feature, support vector machine

## Abstract

Rail corrugation appears as oscillatory wear on the rail surface caused by the interaction between the train wheels and the railway. Corrugation shortens railway service life and forces early rail replacement. Consequently, service can be suspended for days during rail replacement, adversely affecting an important means of transportation. We propose an inspection method for rail corrugation using computer vision through an algorithm based on feature descriptors to automatically distinguish corrugated from normal surfaces. We extract seven features and concatenate them to form a feature vector obtained from a railway image. The feature vector is then used to build support vector machine. Data were collected from seven different tracks as video streams acquired at 30 fps. The trained support vector machine was used to predict test frames of rails as being either corrugated or normal. The proposed method achieved a high performance, with 97.11% accuracy, 95.52% precision, and 97.97% recall. Experimental results show that our method is more effective in identifying corrugated images than reference state-of the art works.

## 1. Introduction

Subways and trains are essential for transportation. Consequently, failures in railways can severely affect the economy, transportation, and living. Serious wear of rails increases the risk of accidents and maintenance costs for rail replacement and grinding operations. In particular, rail corrugation forces premature rail replacement, incurring high costs. The service life of railways can be reduced by rail corrugation and its indicators, such as cracks on the rail surface, rolling contact fatigue, and spalling of the rail head. Statistically, approximately 40% of all rails are prone to developing corrugation [1], thus being one of the most serious and expensive problems in the railway industry.

Corrugation is oscillatory wear on the running surface of a rail caused by long-term interactions between vehicle wheels and rails [2]. Corrugation can affect wheel–rail and vehicle–track interactions and cause noise and vibration in both straight and curved tracks, especially in metro systems. The noise and vibration generated in a moving train is mainly caused by rail corrugation, initially causing discomfort to passengers. As corrugation worsens, increasing vibrations result in the loosening and deterioration of rail components such as fasteners, sleepers, and ballasts. Moreover, corrugation affects the lives of people settling around railway lines owing to the loud noise.

The reliability, safety, and continuation of rail transportation service require regular inspection [3]. Accurate inspection of railways and quantification of their current condition are essential to determine measures for reducing noise and vibration and ensuring their effectiveness. Visual railway inspection by trained staff is the conventional approach for detecting rail surface defects [4]. However, this method is labor intensive, time consuming, subjective, and often inaccurate. To alleviate these problems, automatic inspection is required in practice [5]. The corrugation analysis trolley of RailMeasurement (Stadthagen, Germany) relies on acceleration measurements for inspection and has been widely used in the railway industry. Recently, novel measurement systems using chord measurements or image processing have been developed [6,7].

In this study, an efficient automatic method for rail corrugation detection is proposed. In the previous methods, human interaction was required. So, finding experts in the field was needed. Human error may occur during long repetitive work, and the time cost is high. Hence, by using computer vision-based method there is significant advantage with respect to these issues. The main aim of this research was two-fold: (i) to develop an automatic corrugation classification method based on voice activity detection features, and (ii) to apply Gaussian approximation for subpixel-level estimation for corrugation wavelength calculation.

The remainder of this paper is organized as follows. In Section 2, we discuss related work. In Section 3, we detail the proposed inspection method based on computer vision. Image acquisition is described in Section 3.1, and corrugation identification is detailed in Section 3.2. Experimental results are reported in Section 4, and we draw conclusions in Section 5.

## 2. Related Work

Various methods for automatic corrugation detection based on image processing have been proposed. Although power spectral density analysis has been used to this end, the resulting feature vector has high dimensionality. Other methods use ultrasonic-based defect detection [8], eddy current pulsed thermography [9,10], visual inspection [11], digital image processing [3], and deep convolutional neural networks [12]. In addition, many studies have demonstrated the application of visual inspection for rails using track-profile measurements [13], gauge measurements [14], bogi block keys [15], and fastening monitoring [16,17,18,19].

Various industrial applications for defect inspection have been devised [20,21,22,23]. Remarkably, Li et al. [20] proposed a corrugation identification system based on local frequency features of a segmented track image. First, the rail track was segmented in a captured image, and each column of the rail track was represented by local features. Then, they used a support vector machine (SVM) for corrugation line recognition and monitoring the number of successive (aggregated) corrugated lines for labeling corrugated intervals. However, the dataset used for training was imbalanced because it contained 200 corrugated rail images and 800 normal rail images. In addition, the methods used to prevent overfitting, due to the small and imbalanced dataset, were not reported. In contrast, we convert rail corrugation identification into an image classification problem. Then, a trained classifier is used to determine whether the captured rail image shows corrugation, the wavelength of which is also estimated. Another method was introduced by Mandriota C. [4], a corrugation identification system based on extracting texture features such as Gabor filter, Wavelet transform, and Gabor wavelet transform, then plugged into a K-nearest neighbor classifier. In this method they used a very small amount of data (around 200 images) for training, with 512 pixels of resolution, and similarly for testing. If this was applied to larger images, the method would be slow as it uses Wavelet transform along with a Gabor filter.

## 3. Proposed Method

The proposed inspection method based on computer vision has four main steps: (i) image acquisition; (ii) preprocessing comprising rail-track extraction and 2D-to-1D pixel projection; (iii) feature extraction comprising extraction of entropy, static harmonic feature, peak distance variance, sum of peak prominence, variance of peak prominence, variance of peak width, and sum of peak width features from the vectorized rail image; and (iv) a feature vector used to train an SVM with quadratic, and Gaussian kernel functions to optimize the identification performance.

### 3.1. Image Acquisition

**Figure 1:**
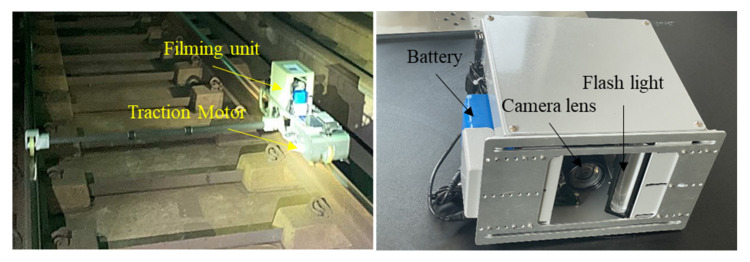
Setup of the image acquisition system used in this study.

Images and videos are collected using an image acquisition system, which consists of commercially available off-the-shelf components, mainly including a line-scan camera, light source, ambient light sensor, and Gigabit Ethernet interface (frame grabber). The line-scan camera (model raL2048–48gm; Basler, Ahrensburg, Germany) has a resolution of 2048 × 2048 pixels and a maximum line rate of 51 kHz, and the computer–camera frame grabber captures rail images. The line-scan camera is calibrated at 2048 lines per second with exposure time of 0.1–0.14 ms facing vertically down the rail track, as shown in Figure 1.

### 3.2. Corrugation Identification

The acquired data are preprocessed for rail corrugation identification. Preprocessing focuses on creating multiscale images and rail track extraction, and corrugation identification comprises feature extraction and classification.

During acquisition, frames may contain additional components, such as connectors and slippers, in addition to the target rails. Hence, the rail track should be segmented to crop the frame for proper inspection. Considering that some rail corrugation lines may be segmented into various frames by the line-scan camera, we concatenate frames with different scales. Multiscale concatenation can facilitate the detection of corrugation with longer waves that extend over multiple frames, and the rail head can be extracted from multiscale frames.

#### 3.2.1. Overview of Proposed Method

**Figure 2:**
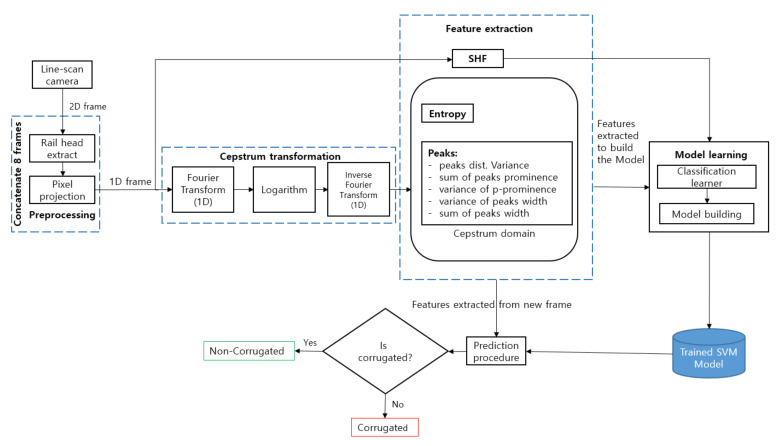
Block-diagram of the proposed method.

The proposed method for rail corrugation detection involves two main processes: (i) building a prediction model, and (ii) applying the model for corrugation detection in new frames. The predictive model is constructed by collecting all the frames required for training in advance. A classification learner is used to build the corrugation classifier. Model building results in a predictive model based on an SVM that predicts the presence of corrugation in previously unseen frames. Both model building and prediction use rail images as inputs extracted following the procedure depicted in Figure 2.

#### 3.2.2. Preprocessing

**Figure 3:**
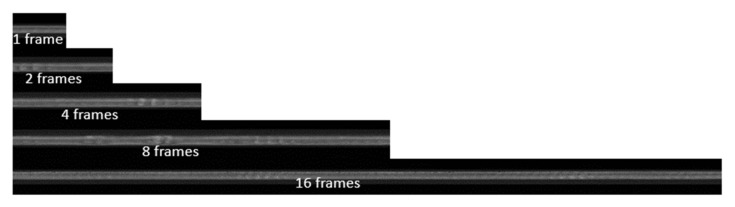
Frames with a scale factor 1, 2, 4, 8, and 16.

Multiscale frames with scale factors of 1, 2, 4, 8, and 16 are concatenated (Figure 3), where a scale factor of *k* (=1, 2, 4, 8, 16) refers to the concatenation of *k* consecutive frames. As corrugation may exist in long rail sections, small images may cut corrugation waves, hindering detection. On the other hand, having a very large-scale image may include a mixture of normal and corrugated rail sections, decreasing the accuracy of corrugation localization. We experimentally determined that a scale factor of 8 provided the best identification results (Table 1).

**Figure 4:**
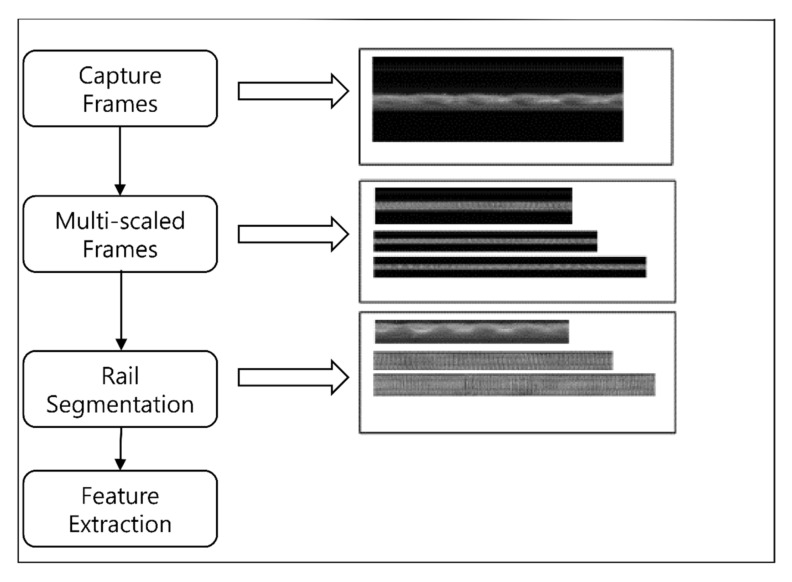
Overview of the preprocessing system.

For rail head extraction, two assumptions are made. First, the rail area is generally fixed in width and located in the middle of the captured image (symmetry). Second, the rail head has a higher average brightness than other rail components. Considering these two assumptions, we apply defect localization based on the projection profile [20] for rail extraction (Figure 4). This algorithm is known as track extraction based on projection profile (TEBP).

For rail head image projection, we vectorize the 2D image by calculating the mean pixel intensity of each column before feature extraction.

#### 3.2.3. Features

**Figure 5:**
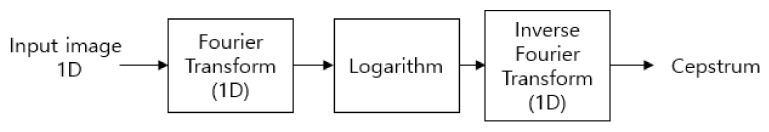
Cepstrum transformation.

We extract features in the cepstral domain or the frequency domain. The cepstral domain is adopted because the cepstrum provides features that are independent of pixel amplitude variations (i.e., scale invariance) or grayscale changes, increasing the robustness against illumination variations [24]. As a Fourier transform is used, these features are also independent of translational shifts [24]. The cepstrum is defined as the inverse Fourier transform of the log-magnitude (Fourier) spectrum [25], as illustrated in Figure 5.

(1) $$c\left(m\right)\;=\;\frac{1}{M}\overset{M\;-\;1}{\underset{m\;=\;0}{\sum }}log\left|S\left(m\right)\right|e^{\frac{j2\pi }{M}m}$$

where *c*(*m*) is the Fourier spectrum of the 1D input frame and *n* is the discrete frequency index. The cepstrum via the cosine transform is defined as the Fourier transform that can be replaced with the cosine transform to reduce computational complexity.

Therefore, Equation (2) is the discrete cosine transform to represent the frequency domain as the cepstrum domain, obtaining features in both the cepstrum and frequency domains.

(2) $$c\left(m\right)\;=\;\frac{1}{M}\overset{M\;-\;1}{\underset{m\;=\;0}{\sum }}log\left|S\left(n\right)\right|\cos\left(\frac{2\pi }{M}m\right)$$

As features, we first use the entropy to study spectral signal irregularity and changes over a range of frequencies on the image waves [26]. Entropy is a useful feature in distinguishing between corrugated and normal surfaces on the rail head. A high entropy reflects smooth or normal rail surfaces, and a low entropy reflects corrugated surfaces. *Entropy H* is derived using the following equations, where *P*(*m*) is the probability distribution of the cepstrum transformation of the 1D image expressed as

(3) $$P\left(m\right)\;=\;\frac{c\left(m\right)}{\sum_{i}c\left(i\right)}$$

(4) $$H\;=\;\overset{N\;-\;1}{\underset{n\;=\;0}{\sum }}p\left(m\right)\log p\left(m\right)$$

We also use static harmonic features, which are common in voice activity detection [27]. As corrugation appears physically as periodical sinusoidal waveforms (sound waves), we use static harmonic features to characterize corrugation and distinguish it from normal surfaces. To obtain the static harmonic features, power spectrum *x*(*j*) of the observed the input 1D image is first calculated by taking the Fourier transform to determine log power spectrum *y*(*j*) of the input signal, as shown in Equation (1). Then, spectrum *y*(*j*) is converted into cepstrum *p*(*i*) by using the discrete cosine transform.

(5) $$p\left(i\right)\;=\;\underset{j}{\sum }M\left(i,j\right).y\left(j\right)$$

where *M*(*i,j*) is the matrix for the discrete cosine transform and *i* indicates the bin number of the cepstral coefficients. Finally, *p*(*i*) is converted back into a log power spectrum using the inverse discrete cosine transform to obtain the linear power spectrum.

(6) $$v\left(j\right)\;=\;\underset{j}{\sum }M{\left(j,i\right)}^{-1}.p\left(i\right)$$

(7) $$w\left(j\right)\;=\;\exp\left\{v\left(j\right)\right\}$$

**Figure 6:**
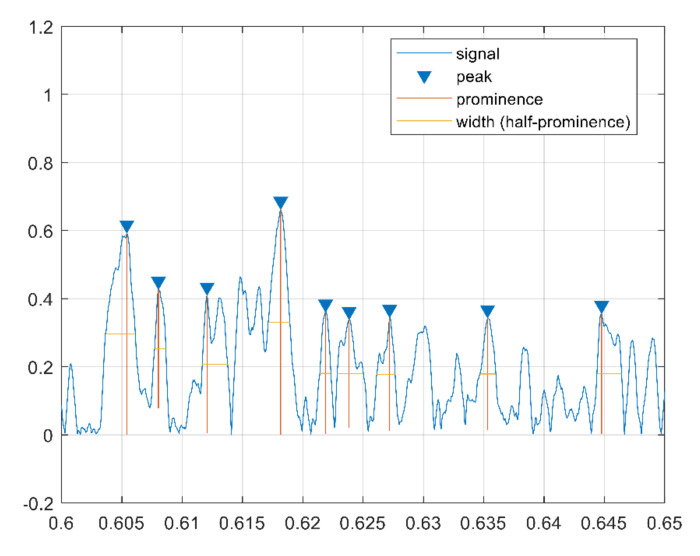
Cepstrum peak features for corrugated sample.

**Figure 7:**
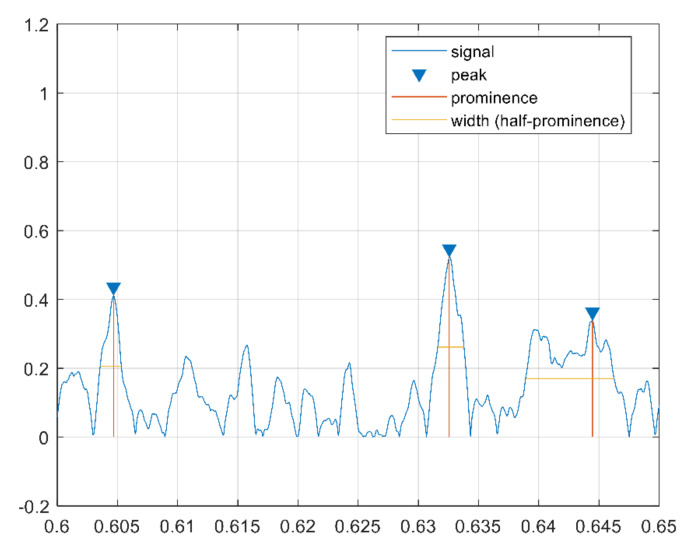
Cepstrum peak features for a noncorrugated sample.

The cepstrum peaks are also used as features in the form of variance of peaks distance, sum of peak prominence, variance of peak width, sum of peak width, and variance of peak prominence. These features are extracted in the cepstrum domain and reflect clear differences between corrugated and normal rails. In fact, a corrugated rail has higher peaks, larger prominence, and smaller width than a normal rail, as shown in Figure 6 and Figure 7, respectively.

**Figure 8:**
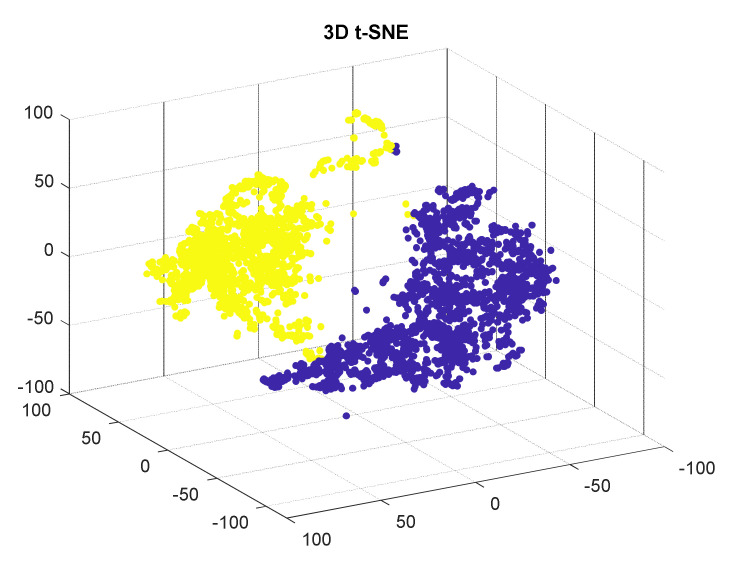
3D visualization of some corrugated samples in cyan, and noncorrugated samples in red, after using t-SNE.

Visualizing a dataset in the feature space allows validation of the effectiveness of the selected features for clustering the target classes. To facilitate visualization, a 3D plot of t-Distributed Stochastic Neighbor Embedding is used. (t-SNE) is a (prize-winning) technique for dimensionality reduction that is particularly well suited for the visualization of high-dimensional datasets (Figure 8). Features were extracted from 1665 corrugated samples and 1831 normal samples.

#### 3.2.4. Classifier

SVMs have been used in many real-world applications such as image classification, bioinformatics, and handwritten character recognition. Although linear SVMs are commonly used, nonlinear SVMs are also available and used depending on the application. The original feature space can be mapped onto a higher-dimensional feature space where the training set is separable by an SVM. We evaluated two kernel functions, quadratic and Gaussian, and experimentally determined that the Gaussian kernel provided the best classification results to detect corrugation.

#### 3.2.5. Frequency Estimation Using Subpixel Analysis

Peak frequency estimation in images is usually performed at the pixel level. However, subpixel-level estimation results in higher accuracy. Fisher [28] compared subpixel-level estimation methods, including Gaussian approximation, linear interpolation, and center-of-mass subpixel estimation. The Gaussian approximation has obvious benefits, as the noise level or stripe width decreases compared with other methods, thus being suitable for our application. We use the Gaussian approximation considering the three highest contiguous intensity values around an observed stripe peak. We assume that the shape of the observed peak fits a Gaussian curve. The subpixel location of peak $\underline{P}$ is given by Equation (8), where *a*, *b*, and *c* represent the intensity of pixels *x* − 1, *x*, and *x* + 1, respectively. Intensity *b* is the highest at location *x*, and intensities *a*, *b*, and *c* are in the range 0-255. A lookup table can be used for logarithm calculations [28].

(8) $$\underline{P}\;=\;x\;-\;0.5\;\times \;\frac{ln\;ln\;\left(c\right)\;-\;ln\;ln\;\left(a\right)\;}{ln\;ln\;\left(a\right)\;+\;ln\;ln\;\left(c\right)\;-\;2\;ln\;ln\;\left(b\right)\;}$$

## 4. Experimental Results

### 4.1. Dataset Preparation

The rail data were collected from seven stations in subway lines as video streams. From each video stream, eight frames were concatenated and labeled to build the dataset, obtaining 5645 images, with 3025 showing normal rails and 2620 showing corrugated rails. Some corrugation patterns were consistent along the entire sample image, while others showed strong corrugation followed by weak corrugation or vice versa, causing irregular corrugation patterns. The dataset also contains ground samples that will be included in future work once the data scarcity of ground samples is overcome.

Both corrugated and normal samples were shuffled, and the dataset was split into 60% for the training set and the remaining 40% for the test set. As each frame has a resolution 2048 × 2048 pixels, concatenating eight frames results in 2048 × 16,384 pixels. Each frame covers 16.05 cm, and thus the eight frames cover a rail length of 1.28 m.

### 4.2. Multiscale Classification

We evaluated four models. Models 1, 2, 3 and 4 were trained with 2, 4, 8 and 16 concatenated frames, respectively. Model 3 achieved the best results among the evaluated models, as reported below.

### 4.3. Confusion Matrix

**Figure 9:**
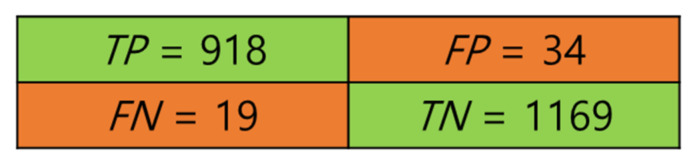
Confusion matrix of the Gaussian kernel SVM model.

The SVM model, along with other models such as Random Forest and KNN, were all trained and, according to the validation accuracy, SVM had the best score (see Table 2). Both SVM with a Gaussian kernel and quadratic kernel had the best accuracy for our dataset (see Table 3). The trained models were evaluated on the test-set (2258 samples). The Gaussian kernel led to a higher performance than the quadratic kernel in terms of accuracy (Equation (9)), precision (Equation (10)), and recall (Equation (11)), as listed in Table 2. Therefore, we used the Gaussian kernel, and Figure 9 shows the confusion matrix for the corrugated and normal classes.

(9) $$Accuracy\;=\;\frac{TP\;+\;TN}{TN\;+\;TP\;+\;FP\;+\;FN}$$

(10) $$Precision\;=\;\frac{TP}{TP\;+\;FP}$$

(11) $$Precision\;=\;\frac{TP}{TP\;+\;FP}$$

### 4.4. Visualization of Classification Results

**Figure 10:**
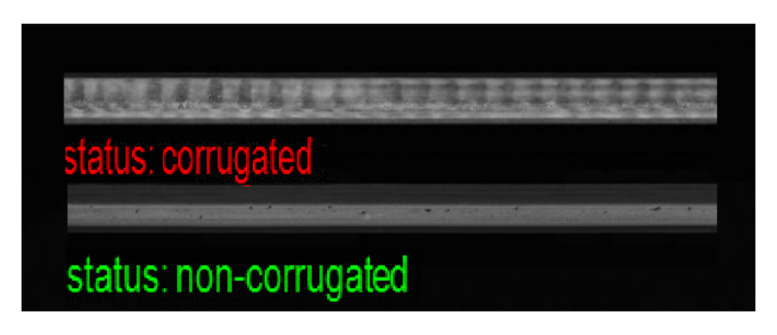
Correctly classified samples.

**Figure 11:**
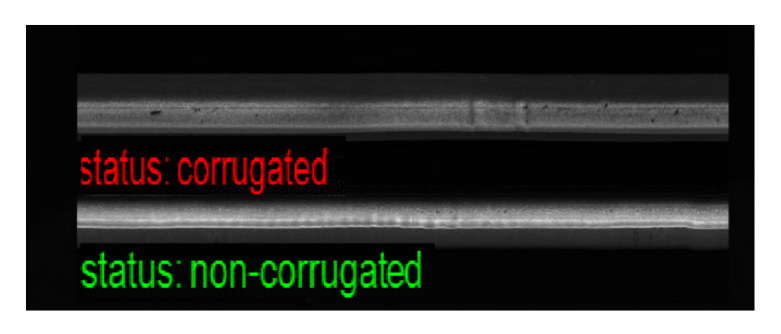
Misclassified samples FP and FN.

Although the proposed method provides high performance for corrugation detection, as shown in Figure 10, there were some misclassified samples, as shown in Figure 11. These samples were challenging to classify owing to small artifacts in the images of normal rails or weak corrugation in the images of corrugated rails.

### 4.5. Sample Frequency Estimation

**Figure 12:**
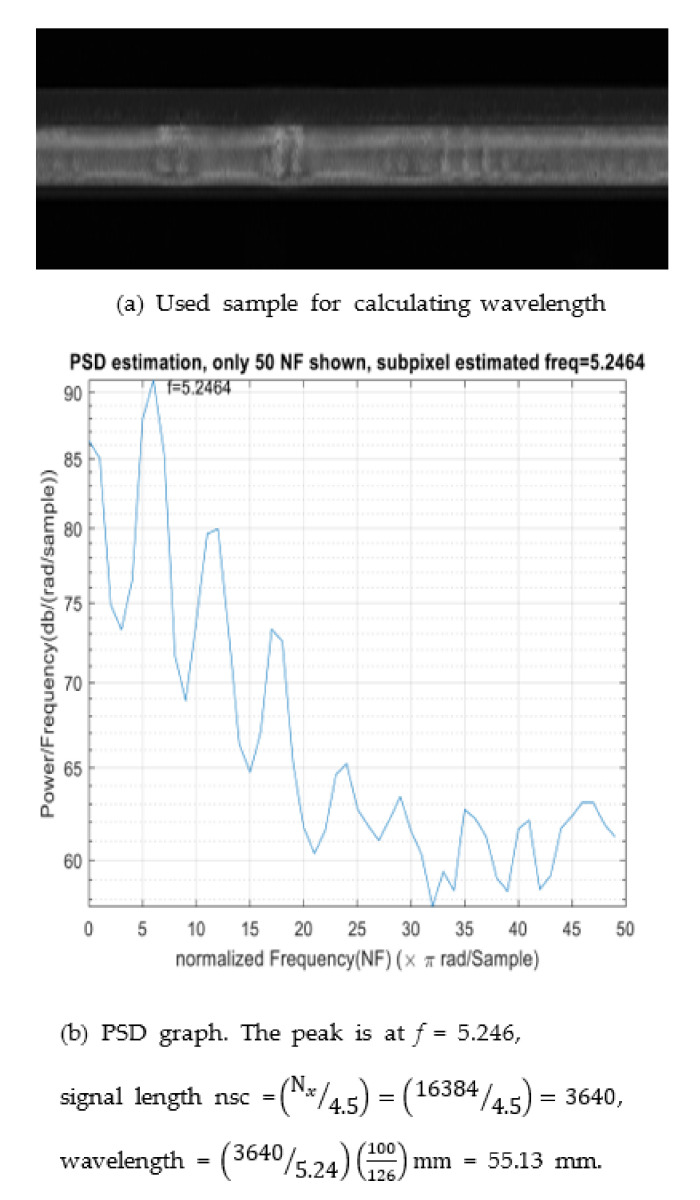
Visualization of Frequency Estimation using Power Spectrum.

For the power spectral density analysis, we obtained the peak frequency component from a frame with 16,384 × 2048 pixels (length × width) Figure 12a. The corresponding power spectrum is shown in Figure 12b, and the peak frequency is *f* = 5.246 Hz, which is the dominant frequency in the image. Subpixel-level frequency estimation was computed using the algorithms described in Section 3.2.5 and performed along with corrugation wavelength calculations in different positions (Table 4). By using a ruler, 1 cm of the rail surface was found to contain approximately 126 pixels, corresponding to 10/126 mm/pixel. This value slightly changed depending on the distance between the lens and rail surface.

### 4.6. Performance Comparison with Related Methods

Performance comparison between our method and previous methods is given in Table 5. The experimental results show that our method outperforms the previous methods. Li’s method [20] and Mandriota’s method [4] have been used for comparison with proposed method to evaluate performance. Li’s method applies FFT for feature extraction and the SVM model for corrugation line identification. Mandriota’s method applies a Gabor filter for feature extraction and a K-nearest neighbor classifier. Our proposed method shows higher precision, higher recall, and higher accuracy rate.

## 5. Conclusions

We proposed a rail inspection method based on computer vision to detect corrugation and estimate the corrugation wavelength in rail heads. The proposed method is based on handcrafted features and SVM for corrugation detection, while subpixel frequency estimation is used to determine the corrugation wavelength. Experimental results show that the proposed method achieves higher performance in the identification of rail corrugation than similar methods. We compared performance regarding precision, recall and accuracy. Our method achieved 97.97% recall, 97.11% accuracy, and 95.52% precision. In addition, the proposed method successfully identified corrugated images with a low false negative rate below 2%. The high recall rate can contribute to avoiding accidents due to misidentification of corrugated rails as normal rails. After detecting the corrugated samples, the proposed method provides the corrugation wavelength by estimating the peak frequency using subpixel analysis. All samples used as test images in this study were acquired from a subway line, and the corrugation wavelength ranged from 20.61 to 55.59 mm. In future work, we will investigate image acquisition from newly replaced rails for early analysis of corrugation based on computer vision and image processing. In addition, we will supplement the algorithm for distinguishing ground rail images from corrugated rail images.

## Figures and Tables

**Table 1:** Performance comparison between 2, 4, 8, and 16 frames using our proposed method.

Model	*P* (%)	*R* (%)	*A* (%)
Model 1	97.11	95.52	97.97
Model 2	92.00	91.00	91.50
Model 3	91.00	90.00	90.50
Model 4	94.53	92.30	94.12

**Table 2:** Performance comparison between different models.

Model	Accuracy
SVM	97.6%
Random Forest	94.1%
KNN	96.2%
Decision tree ensemble	96.6%

**Table 3:** Performance comparison between SVM-Gaussian and SVM-Quadratic.

Model	*P* (%)	*R* (%)	*A* (%)
SVM Gaussian	95.52	97.97	97.11
SVM Quadratic	92.45	95.51	94.64

**Table 4:** Wavelength and subpixel frequency estimation of different testing sites.

Site	Peak Freq. (Hz)	Wavelength (mm)
Test site 1	7.12	39.47
Test site 2	6.75	41.65
Test site 3	4.97	55.91
Test site 4	8.10	30.17

**Table 5:** Performance comparison between our method and the baselines.

Method	*P* (%)	*R* (%)	*A* (%)
Qingyong Li’s method	95.37	95.50	94.30
Mandriota C.’s method	73.46	95.50	92.20
Our method	95.52	97.97	97.11

## Data Availability

Not applicable.
